# Evaluation of Dry Eye Parameters Among Electronic Cigarette Smokers

**DOI:** 10.7759/cureus.62148

**Published:** 2024-06-11

**Authors:** Rui Ping Chew, Kevin Kwan Joo Ern, Julieana Muhammed, Akmal Haliza Zamli

**Affiliations:** 1 Department of Ophthalmology and Visual Science, School of Medical Sciences, Universiti Sains Malaysia, Kota Bharu, MYS; 2 Department of Ophthalmology, Hospital Tengku Ampuan Afzan, Kuantan, MYS

**Keywords:** meibomian gland dysfunction, smoking, e-cigarette, electronic cigarette smokers, dry eye

## Abstract

Introduction

The emergence of electronic cigarettes as the “healthier” version of smoking has been popular, especially among young adults. However, knowledge about the potential effects of e-cigarettes on ocular structures is scarce.

Objective

To compare the mean change in dry eye parameters between e-cigarette smokers and non-smokers and to correlate between dry eye parameters with device power output.

Methodology

A cross-sectional, single-visit study was conducted involving 85 e-cigarette smokers and 85 non-smokers. All participants were evaluated on dry eye parameters, including the Ocular Surface Disease Index (OSDI) score, tear meniscus height (TMH), tear break-up time (TBUT), cornea fluorescein staining, and Schirmer’s I test. The mean change in dry eye parameters was compared between e-cigarette smokers and non-smokers. The correlation between dry eye parameters with device power output was analyzed.

Results

Specifically, 32.94% of e-cigarette smokers were found to have a TMH <0.2 mm, compared to only 5.88% of non-smokers (p<0.001). A significant change in mean TBUT was found between the e-cigarette smokers (10.41+2.65 seconds) and non-smokers (12.66+3.14 seconds, p<0.001). The lower mean Schirmer’s I test was found among e-cigarette smokers (12.75+7.24 mm, p<0.001). No significant change in the median OSDI score and corneal fluorescein staining. The OSDI score and device power output were found to have a significant positive correlation (p=0.003). There was a significant association between dry eye signs and device power output, including TMH (p=0.047), TBUT (p=0.002), Schirmer’s I test (p<0.001), and corneal fluorescein staining (p<0.001).

Conclusion

Electronic cigarette smokers are prone to develop dry eyes. Concern should be raised on the risk of electronic cigarette use on ocular health, and regulation on e-cigarette ban should be revisited.

## Introduction

Dry eye disease (DED), also known as dry eye syndrome, is defined by Tear Film and Ocular Surface Society (TFOS) Dry Eye Workshop II as a multifactorial disorder of the ocular surface, characterized by eye discomfort and visual disturbance secondary to the loss of homeostasis and instability of the tear film, hyperosmolarity, and inflammation of the ocular surface [1]. An electronic cigarette, also known as an e-cigarette and electronic nicotine delivery system, is a battery-operated device containing a cartridge filled with liquid nicotine and/or other chemicals, producing vapor containing nicotine [2].

The Beaver Dam Eye study in 2000 showed that the prevalence of DED was 14.4% among the population aged between 48 and 91 years, and a twofold increase in the incidence of DED was found among smokers [3]. E-cigarette usage has been on the rise in recent years. It has gained popularity among smokers as it has been presumed as a “healthier” version of tobacco cigarette smoking with fewer side effects [4]. A rise in the number of e-cigarette users from 1.5% in year 2011 to 20.8% in year 2018 was previously reported among high school students in the United States [5]. A similar pattern of e-cigarette usage rise was observed among Malaysians. The prevalence of e-cigarette users among adults in Malaysia was reported to be 0.8% in 2011 by the Global Adult Tobacco Survey, and an increasing trend of e-cigarette smokers to 4.9% was found by the National Health & Morbidity Survey 2019 [6,7]. However, studies about the effect of electronic cigarettes on the ocular surface have been very limited.

DED with moderate to severe eye dryness and disturbed tear film integrity was reported among electronic cigarette users [4]. It is believed that the toxic and irritative contents of cigarette smoke are linked with ocular surface epithelium damage when it comes into direct contact with the ocular surface. Chronic exposure to cigarette smoke resulted in inflammatory conjunctival reactions similar to those exposed to chronic irritation [8]. Additionally, electronic cigarette smoking was shown to affect the loss rate of eyelid meibography with e-cigarette smoking duration [9].

The rationale for this study is based on the significant prevalence of DED associated with electronic cigarette use. This is due to direct contact with the irritative and toxic contents, as well as the free-radical components of e-cigarettes, which cause an unstable precorneal tear film. Identifying DED among electronic cigarette users aims to provide new insight into whether electronic cigarettes should not be considered as harmless as it was marketed and whether stricter regulation of e-cigarettes and their marketing should be enforced.

## Materials and methods

This is a cross-sectional study conducted between January 2021 to June 2022. Electronic cigarette smokers aged between 18 and 45 years old who were attending quit smoking clinics of Hospital Tengku Ampuan Afzan (HTAA) and Hospital Universiti Sains Malaysia (HUSM) or electronic cigarette retail shops around Kuantan, Pahang, and Kubang Kerian, Kelantan, and have smoked daily for at least one year were recruited. Those with anterior segment pathologies such as keratoconus, cornea scar, DED, history of cornea, refractive and glaucoma surgery, regular contact lens wearer, using any eye drops (such as anti-glaucoma and anti-inflammatory medication), dual smokers (conventional tobacco and electronic cigarettes), ex-smoker who has smoked more than 100 cigarettes in his or her lifetime, and ex-occasional smoker but quitted tobacco smoking for less than six months were excluded from this study. Written consent was taken.

The electronic cigarette smokers were asked about the total duration of electronic cigarette smoking quantified in years, e-cigarette device power output, and the number of puffs taken per day. If multiple e-cigarette devices were used, the most frequently used device reading was taken. Information regarding the number of puffs taken per day was recorded. All participants then answered the Ocular Surface Disease Index (OSDI) questionnaire. Tear meniscus height (TMH) measurement was done using a slit lamp biomicroscope’s illuminated slit width by setting the slit horizontally in alignment with the lower lid margin. The slit width was adjusted until it appears to match the height of the inferior tear meniscus centrally in alignment with the pupil. Tear break-up time (TBUT) was measured by using a fluorescein sodium ophthalmic strip, which was wet with non-preserved artificial tear eye drops. Assessment of cornea fluorescein stain was done, and the National Eye Institute (NEI) corneal grading system was used. The subject was asked to rest for five minutes, followed by Schirmer’s I test.

All data were analyzed using the IBM Statistical Package for Social Sciences (SPSS, version 26.0; IBM SPSS Statistics for Windows, Armonk, NY) for Windows. A comparison of the frequency and percentage of TMH between electronic cigarette smokers and non-smokers was done with the Pearson chi-square test. The Mann-Whitney U test was applied to compare the median changes of the OSDI score and corneal fluorescein staining. Other dry eye signs, which include TBUT and Schirmer’s I score, were analyzed using the independent T-test. Correlation analyses between the OSDI score and device power output, as well as dry eye signs (TBUT, corneal fluorescein staining, and Schirmer’s I test) and device output, were performed using the Pearson correlation test. Correlation analysis between TMH and device power output was measured using Spearman’s rank correlation. P-value <0.05 was considered statically significant for all statistical analyses.

This study was reviewed and approved by the Medical Research and Ethics Committee (MREC) of the Ministry of Health Malaysia (IRB Approval Number: NMRR-20-2446-55611). The approval was granted on December 7, 2020. Additionally, this study received ethical approval from the Jawatankuasa Etika Penyelidikan Manusia (JEPeM), School of Medical Sciences, Universiti Sains Malaysia, on March 31, 2021 (JEPeM Code: USM/JEPeM/21010045).

All procedures performed in this study involving human participants were in accordance with the ethical standards of the institutional and/or national research committee and with the 1964 Helsinki Declaration and its later amendments or comparable ethical standards. Informed consent was obtained from all individual participants included in the study. Participant confidentiality and anonymity were strictly maintained throughout the research process.

## Results

A total of 85 electronic cigarette smokers and 85 non-smokers were recruited for this study. The mean age of this study was 29.0+7.4 years. Among the electronic cigarette smokers, the mean age was 29.3+8.1 years, whereas the mean age for the non-smoker group was 28.64+6.7 years. The characteristics of electronic cigarette consumption are presented in Table 1.

**Table 1:** The characteristics of electronic cigarette consumption Abbreviation: n, number, SD, standard deviation

Variables Mean (SD)	Electronic cigarette smokers, n=85
Smoking duration (years)	2.2 (1.0)
E-cigarette consumption (puffs taken per day)	221.94 (81.64)
Device power output (wattage)	23.2 (6.1)

Specifically, 32.94% of electronic cigarette smokers were found to have a TMH <0.2 mm, compared to only 5.88% of non-smokers with TMH <0.2 mm (p<0.001). There was a statistically significant change in mean TBUT between the electronic cigarette smokers and non-smokers with a p-value<0.001. No difference was recorded in the median change in the OSDI score (p=0.101) and corneal fluorescein staining score (p=0.061) for e-cigarette smokers and non-smokers. A significant difference was found in the mean Schirmer’s I test between electronic cigarette smokers and non-smokers (p<0.001). The mean and median change of dry eye parameters are illustrated in Table 2.

**Table 2:** Comparison of the mean and median change of the OSDI score, TMH, TBUT, corneal fluorescein staining score, and Schirmer’s I test between electronic cigarette smokers and non-smokers ^a^ Pearson chi-square test; ^b^ Mann-Whitney U test; ^c^ Independent T-test p<0.05 was considered statistically significant. Abbreviation: CI, confidence interval; df, degrees of freedom; n, number; IQR, interquartile range; OSDI, Ocular surface disease index; SD, standard deviation; TBUT, tear break-up time; TMH, tear meniscus height

Variables	Electronic Cigarette Smokers, n=85	Electronic Cigarette Smokers, n=85 (%)	Non-smokers, n=85	Non-smokers, n=85 (%)	Mean Difference, 95% CI)	Test Statistic (df)	p-value
TMH <0.2 mm	28	32.94	5	5.88	-	19.89(1)	<0.001^a^
TMH >0.2 mm	57	67.06	80	94.11			
OSDI score, Median (IQR)	0.00 (0.00-6.25)		0.00 (0.00-2.08)		-	4084	0.101^b^
Corneal fluorescein staining score, Median (IQR)	0.00 (0.00-0.00)		0.00 (0.00-0.00)		-	3948.5	0.061^b^
TBUT (seconds), Mean (SD)	10.41 (2.65)		12.66 (3.14)		-2.25 (-3.13, -1.37)	-5.043 (168)	<0.001^c^
Schirmer’s I test (mm), Mean (SD)	12.75 (7.24)		20.02 (7.98)		-7.27 (-9.58, -4.96)	-6.220 (168)	<0.001^c^

The correlation of dry eye parameters with device power output is shown in Table 3. A weak positive correlation was found between the OSDI score and device power output (p=0.003). Both TMH (p=0.047) and TBUT (p=0.002) were reduced with the increase in electronic cigarette device power output. A weak positive correlation was found between corneal fluorescein staining and device power output (p<0.001). A moderate negative correlation was found between Schirmer’s I test and device power output (p<0.001).

**Table 3:** Correlation between dry eye signs (TMH, TBUT, corneal fluorescein staining, and Schirmer’s I test) and device power output in electronic cigarette smokers ^a^Spearman rank correlation; ^b^Pearson correlation P-value <0.05 was considered statistically significant. Abbreviation: r, correlation coefficient; SD, standard deviation; TBUT, tear break-up time; TMH, tear meniscus height

Variables	Device Power Output (wattage) r value	p-value
TMH	-0.216	0.047^a^
TBUT (sec)	-0.330	0.002^b^
Corneal Fluorescein Staining	0.378	<0.001^b^
Schirmer’s I test (mm)	-0.488	<0.001^b^

## Discussion

A very limited number of female e-cigarette smokers could be found in the study centers, and most of them were not willing to admit themselves as e-cigarette smokers. We postulated this could be due to the social gendered stigma of the population towards female smokers, such as loss of feminine status and perception as out of control [10].

Challenges are faced in the accurate quantification of electronic cigarette use due to the variations in the number of puffs taken per session each day, duration of each session, wattage used, e-liquid consumed, and nicotine concentration [11]. In the present study, the calculation of device power output was based on the most frequently used electronic cigarette device and power output that was commonly set daily, instead of the cumulative power output throughout the smoking duration due to difficulty in the generation of the total power output used in e-cigarette devices and user behavior heterogenicity with multiple devices.

Kalayci et al. found a statistically significant difference in the mean OSDI score between electronic cigarette smokers, with a mean score of 28.60+6.54 compared to 15.16+7.23 among non-smokers [9]. However, we recorded a much lower median OSDI score for both e-cigarette smokers and non-smokers groups. Younger adults were reported to be less likely to seek medical consultation, and in the reproductive age group of 21-39 years old, the lowest male-to-female consultation ratio of 0.40 was reported [12]. The lack of health concerns and less likelihood to report minor symptoms could possibly be the reasons for the low OSDI score obtained among male e-cigarette smokers in this study. Another postulation for the discrepancy in the OSDI score between our study and that of Kalayci et al. is the difference in e-cigarette smoking duration. Our study recruited e-cigarette smokers who smoked for at least one year, which is comparatively shorter in duration than those recruited by Kalayci et al., who were taking e-cigarettes for at least three years [9]. DED symptoms might be too subtle to be quantified objectively by subjects who had a shorter electronic cigarette usage period.

E-cigarette smokers are at higher risk of dry eye with reduced tear volume among the electronic cigarette smokers, as manifested through TMH and Schirmer’s I test. A similar conclusion was drawn by Md Isa et al. with the median TMH of 0.203 (0.193-0.226) mm among the vapers compared to 0.235 (0.210-0.254) mm in the control groups [4]. The presence of free radicals from electronic cigarettes that mediate lipid peroxidation is postulated to damage the lipid layer of the tear film, causing a lower TMH [13].

Several studies on tobacco cigarette smokers demonstrated lower Schirmer’s II results among conventional cigarette smokers, suggesting reduced tear production and tear volume with tobacco cigarette exposure [14,15]. Histopathological alteration of conjunctiva such as squamous metaplasia with changes to cornea nerve plexus, resulting in the reduction of conjunctival and corneal sensitivity. Thus, a decrease in basal tear secretion was revealed with the use of tobacco smoking [8,16]. We deduce that similar histopathological changes that happened to tobacco cigarette smokers can occur among electronic cigarette users and thus a reduction in both basal and reflex tear secretion, as manifested in Schirmer’s I test results. To our best knowledge, there is no literature report on Schirmer’s I test among e-cigarette smokers. Our study adds to the literature that electronic cigarette usage reduces both basal and reflex tear secretion and that the lower tear volume will risk for DED.

Our study result substantiates that the hazardous irritants produced by electronic cigarettes, such as carbonyl compounds and volatile organic compounds, result in shorter mean TBUT among electronic cigarette smokers as compared to non-smokers. This is consistent with the study by Md Isa et al. that found a significantly lower median TBUT time among vapers [4]. The direct contact of vapor irritants and the heat generated during the vaporization of e-juice from electronic cigarettes are likely the culprits to cause corneal epithelium damage, in which various research have revealed worsened corneal fluorescein staining among tobacco cigarette smokers compared to non-smokers [8,17].

It has been discovered that, with higher power output and coiling temperature generated, a bigger volume of vapor and a larger amount of aerosol collected mass (ACM) of e-cigarettes, which contain free radicals, carbonyl compounds such as formaldehyde, acetaldehydes and acrolein, volatile organic compounds, and heavy metals, will be released [18-20]. Geiss et al. found that, with an electronic cigarette device power output wattage set in between 10 W and 15 W, a surge in the amount of carbonyl compound emissions up to 10 times was reported compared to a device set at 5 W [20]. We reported a higher OSDI score with the increase in device power output. This is consistent with the report by Md Isa et al. [4]. From these, we can infer that higher device power output potentially impacts the ocular surface negatively with poorer tear film performance, thus resulting in increased dry eye symptoms.

The literature on the relationship between TMH and electronic cigarette power output is scarce. Md Isa et al. illustrated down-trend TMH and TBUT with higher vaping voltage [4]. A significant association between dry eye parameters with power output strengthens the evidence of increased toxic substances generated from e-cigarettes when a higher device power output was used [20]. A device set with higher wattage generates a larger amount of ACM and releases extra free radicals, which play a role in the further destruction of the tear lipid layer through lipid peroxidation [13]. The breakdown of the tear lipid layer may result in evaporative dry eye and manifest as reduced TMH, shorter TBUT, and corneal fluorescein staining.

There are a few limitations in our study. Passive smokers were not excluded from our study. There are other factors, such as occupations related to clerical support, craft work, and technicians with exposure to heat and hot environments, and environmental factors such as air-conditioning, as well as computer and visual display terminal use that may affect dry eye parameters, were not being excluded in the present study. The meticulous selection of the study population has to be formulated when conducting future research on assessing the ocular surface among e-cigarette smokers. The cumulative power output used throughout the total smoking duration was not calculated due to the difficulties in the generation of the total power output of e-cigarettes. The difficulty was faced in the accurate quantification of electronic cigarette use as there are different e-juice nicotine concentrations, e-liquid consumed, duration of each session, and user behavior heterogenicity with multiple devices. Consensus on e-cigarette use intensity measurement has important implications in helping the researchers on improving accuracy in the quantification of e-cigarette use and better understanding of its impacts on health.

## Conclusions

Electronic cigarette smokers are prone to developing dry eye and other ocular issues. While further research is necessary to establish the long-term consequences definitively, current findings indicate that e-cigarette vapor can adversely affect the eyes. This highlights the importance of raising awareness and exercising caution among users. Given these potential risks, there is a strong case for revisiting and possibly strengthening regulations on e-cigarettes.

## References

[1] Craig JP, Nichols KK, Akpek EK (2017). TFOS DEWS II definition and classification report. Ocul Surf.

[2] Puteh SE, Manap RA, Hassan TM (2018). The use of e-cigarettes among university students in Malaysia. Tob Induc Dis.

[3] Moss SE, Klein R, Klein BE (2000). Prevalence of and risk factors for dry eye syndrome. Arch Ophthalmol.

[4] Md Isa NA, Koh PY, Doraj P (2019). The tear function in electronic cigarette smokers. Optom Vis Sci.

[5] Cullen KA, Ambrose BK, Gentzke AS, Apelberg BJ, Jamal A, King BA (2018). Notes from the field: use of electronic cigarettes and any tobacco product among middle and high school students - United States, 2011-2018. MMWR Morb Mortal Wkly Rep.

[6] Ab Rahman J, Mohd Yusoff MF, Nik Mohamed MH (2019). The prevalence of e-cigarette use among adults in Malaysia. Asia Pac J Public Health.

[7] Institute for Public Health, Ministry of Health, Malaysia Malaysia (2020). Volume I: NCDs - Non-Communicable Diseases: Risk Factors and Other Health Problems. https://iku.moh.gov.my/images/IKU/Document/REPORT/NHMS2019/Report_NHMS2019-NCD_v2.pdf.

[8] Satici A, Bitiren M, Ozardali I, Vural H, Kilic A, Guzey M (2003). The effects of chronic smoking on the ocular surface and tear characteristics: a clinical, histological and biochemical study. Acta Ophthalmol Scand.

[9] Kalayci M, Cetinkaya E, Yaprak L, Yigit K, Suren E, Dogan B, Erol MK (2023). Ocular surface assessment and morphological alterations in meibomian glands with non-contact meibography in electronic cigarette smokers. Arq Bras Oftalmol.

[10] Antin TM, Annechino R, Hunt G, Lipperman-Kreda S, Young M (2017). The gendered experience of smoking stigma: implications for tobacco control. Crit Public Health.

[11] Soule E, Bansal-Travers M, Grana R, McIntosh S, Price S, Unger JB, Walton K (2023). Electronic cigarette use intensity measurement challenges and regulatory implications. Tob Control.

[12] Wang Y, Hunt K, Nazareth I, Freemantle N, Petersen I (2013). Do men consult less than women? An analysis of routinely collected UK general practice data. BMJ Open.

[13] Altinors DD, Akça S, Akova YA, Bilezikçi B, Goto E, Dogru M, Tsubota K (2006). Smoking associated with damage to the lipid layer of the ocular surface. Am J Ophthalmol.

[14] Khalil HEM, Aboud SA, Azzab MA (2018). Comparative study between smokers and nonsmokers regarding dry eye. Delta J Ophthalmol.

[15] Bhutia P, Sen S, Nath T, Shamshad MA (2021). The effect of smoking on ocular surface and tear film based on clinical examination and optical coherence tomography. Indian J Ophthalmol.

[16] Narnoli P, Dhasmana R, Khanduri R (2021). Dry eye disease and retinal nerve fiber layer changes in chronic smokers. Indian J Ophthalmol.

[17] Mohidin N, Jaafar AB (2020). Effect of smoking on tear stability and corneal surface. J Curr Ophthalmol.

[18] Goniewicz ML, Knysak J, Gawron M (2014). Levels of selected carcinogens and toxicants in vapour from electronic cigarettes. Tob Control.

[19] Sleiman M, Logue JM, Montesinos VN, Russell ML, Litter MI, Gundel LA, Destaillats H (2016). Emissions from electronic cigarettes: key parameters affecting the release of harmful chemicals. Environ Sci Technol.

[20] Geiss O, Bianchi I, Barrero-Moreno J (2016). Correlation of volatile carbonyl yields emitted by e-cigarettes with the temperature of the heating coil and the perceived sensorial quality of the generated vapours. Int J Hyg Environ Health.

